# Association of shared decision making with inpatient satisfaction: a cross-sectional study

**DOI:** 10.1186/s12911-021-01385-1

**Published:** 2021-01-25

**Authors:** Huiwen Luo, Guohua Liu, Jing Lu, Di Xue

**Affiliations:** 1 Department of Hospital Management, School of Public Health, NHC Key Laboratory of Health Technology Assessment, Fudan University, No. 138, Yi Xue Yuan Road, Shanghai, 200032 People’s Republic of China; 2 Shanghai Medical Ethos Association, Jiangan District, No. 328, Huashan Road, Shanghai, 200040 People’s Republic of China

**Keywords:** Shared decision making, Patient satisfaction, Medical care quality, Tertiary hospitals

## Abstract

**Background:**

We assessed inpatient perceived shared decision making (SDM) and tested the association of SDM with inpatient satisfaction in public tertiary hospitals in Shanghai, China.

**Methods:**

A cross-sectional survey of 2585 inpatients in 47 public tertiary hospitals in Shanghai in July and August 2018 was conducted. We assessed overall SDM and 4 aspects of SDM and tested the factors influencing SDM and the association of SDM with patient satisfaction (patient satisfaction with physician services, medical expenses, outcomes and overall inpatient care), by adopting linear or two-level regression models.

**Results:**

The positive response rate (PRR) and high positive response rate (HPRR) to overall SDM among the inpatients of public tertiary hospitals in Shanghai were relatively high (95.30% and 87.86%, respectively), while the HPRR to “My physician informed me of different treatment alternatives” was relatively low (80.09%). In addition, the inpatients who underwent surgery during admission had higher HPRRs and adjusted HPRRs to overall SDM than those who did not undergo surgery. The study showed that the adjusted high satisfaction rates (HSRs) with physician services, medical expenses, outcomes and overall inpatient care among the inpatients with high level of overall SDM were higher (96.50%, 68.44%, 89.50% and 92.60%) than those among the inpatients without a high level of overall SDM (71.77%, 35.19%, 57.30% and 67.49%). The greatest differences in the adjusted HSRs between the inpatients with and without a high level of SDM were found in inpatient satisfaction with medical expenses and informed consent in SDM. Moreover, 46.22% of the variances in the HSRs with overall inpatient care across the hospitals were attributed to the hospital type (general hospitals vs. specialty hospitals).

**Conclusions:**

Inpatient PRRs and HPRRs to SDM in public tertiary hospitals in Shanghai are relatively high overall but lower to information regarding alternatives. SDM can be affected by the SDM preference of both the patients and physicians and medical condition. Patient satisfaction can be improved through better SDM and should be committed at the hospital level.

## Background

Shared decision making (SDM) involves the participation of both physicians and patients in medical decision making by weighing the available medical evidence and the values and preferences of patients [1, 2]. The aim of SDM is to promote patient autonomy and make informed, patient-centered decisions [3, 4]. SDM consists of the following four elements: two parties (physicians and patients) involved in SDM, participation during the process of decision making by both physicians and patients, information sharing as a prerequisite for SDM, and a treatment decision made and agreed upon by the physician and patient [5]. The core of SDM is that physicians and patients develop the best treatment plan for the patients through discussion with the aim to maximize patients’ benefits [6, 7].

To measure SDM, the API [8], the Perceived Involvement in Care Scale (PICS) [9], the 9-item Shared Decision Making Questionnaire (SDM-Q-9) [10], the Shared Decision Making Questionnaire-physician version (SDM-Q-Doc) [11], and CollaboRATE [12] are often used to assess patients’ information preference, patient involvement in SDM and encouragement from physicians to achieve SDM. However, informed consent, which reflects respect for patient autonomy in special medical care (such as high-risk, costly or considerable out-of-pocket medical care), is not included in current SDM studies. In an era during which new technology is developed very rapidly, informed consent should not be neglected when some new technologies introduce higher risks and financial burden to patients.

In studies of SDM, patient characteristics (e.g., age, education level, and gender) and suitable treatment situations for SDM (e.g., disease characteristics, therapeutic options and availability of scientific evidence regarding the treatment efficacy) are considered potential factors that may affect SDM by some researchers [13, 14]. Additionally, socioeconomic factors may influence patients’ perceptions of SDM [15].

Patient satisfaction is a key measure of the quality of healthcare systems that reflects patients’ experiences and has been added to the performance assessments of hospitals in some countries [16, 17]. Many researchers have studied on the effect of SDM on patient satisfaction, treatment satisfaction, decision satisfaction and trust, timeliness of diagnosis and decisions, necessity of referrals, diagnostic tests and medical treatment, patient adherence to medications and treatment, patient safety, health outcomes, and patient rights and welfare [18–26]. Some studies reported positive results, while some studies indicated that a negative or no relationship exists between SDM and health outcomes [27]. Because physician services represent a basic concern in medical care, medical expenses still impose a great financial challenge to patients, and patients are the least satisfied with medical expenses [28]. Treatment outcomes affect patients’ health and quality of life; thus, determining whether a high level of SDM specifically leads to better patient perceptions of physician services, medical expenses and treatment outcomes is warranted.

## Objectives

The goals of this study were to analyze the status of SDM and the influencing factors of SDM in inpatient care in tertiary public hospitals in Shanghai, and determine whether SDM leads to higher inpatient satisfaction with overall inpatient care, physician services, medical expenses and treatment outcomes.

## Methods

### Data source

A cross-sectional inpatient survey was conducted in 47 tertiary public hospitals (32 general hospitals and 15 specialty hospitals) in Shanghai in July and August 2018. Only three tertiary public hospitals in Shanghai (one mental health center, one hospital specializing in infectious disease, and one hospital with no inpatient care) were excluded from the study. Because 90% of all patients receive medical care at public hospitals in China [29], patient care in public hospitals can generally represent patient care in China.

A random sample of inpatients who had completed their main medical care (e.g., surgeries or therapeutic procedures) was selected from each of the sampled tertiary public hospitals within one workweek. The average number of sampled inpatients per hospital was 55 (52–79). All voluntary investigators, who were mainly senior medical students from major medical colleges in Shanghai, received training regarding the inpatient survey. The survey was conducted via an e-questionnaire administered using iPads. Oral informed consent was obtained before the patients’ participation in the survey.

In the questionnaire survey, data related to inpatient satisfaction, inpatients’ perceived SDM, public hospital type (general vs. specialty), inpatient characteristics [e.g., gender, age, residence (Shanghai vs. non-Shanghai), education, family monthly income (< 5 k, 5 k-, 10 k-, 20 k-, or 50 k yuans)], patients with or without cancer (yes vs. no), having surgery (yes vs. no) and admitting clinical department (e.g., internal medicine, surgery, gynecology, pediatrics, other) were collected.

### Measures

#### SDM scale

Four aspects were used to assess SDM in inpatient care, including “Patients’ information preference”, “Patients’ active involvement in SDM”, “Patients’ perceived encouragement from their physicians to achieve SDM” and “Informed consent”. Of the four aspects, the former two aspects reflected the patients’ desire for autonomy, while the latter two aspects reflected the patients’ perceived autonomy support [20]. The items in the aspects of “Patients’ information preference”, “Patients’ active involvement in SDM”, and “Patients’ perceived encouragement from their physicians to achieve SDM” were based on the API [8], PICS [9], and the SDM-Q-9, SDM-Q-Doc, CollaboRATE and PICS [9–12], respectively. The items in the aspect of “Informed consent” were developed by the authors. Twenty-five experts in medical care quality from Shanghai were consulted regarding all items on the SDM scale. During the consultation, each item related to SDM was rated according to its importance by experts, and a 10-point scale was adopted for the rating (1 for “very unimportant” and 10 for “very important”). If the average score of the importance of an item was equal to or greater than 7, the item was included in the SDM assessment (Additional file 1: Table S1). Finally, 13 items were used in this study to assess the four aspects of SDM in inpatient care (Table 1).

**Table 1:** PRRs and HPRRs to SDM among inpatients in tertiary hospitals in Shanghai

Aspects and items	PRR (%)	HPRRs (%)
**Patients’ information preference**	**97.33**	**90.04**
I should sufficiently understand the effects of the disease(s) that I have on my health	96.43	88.33
The physician should explain to me the purposes of the test(s) and/or examination(s)	98.07	90.95
I believe that getting information about the disease(s) is as important as getting information about the treatment	97.48	90.83
**Patients’ active involvement in SDM**	**93.71**	**85.57**
I asked my physician to explain the treatment alternatives and process in detail	91.78	83.92
I asked my physician to provide treatment recommendations to me	91.10	82.29
I described my disease symptoms to my physician in detail	98.26	90.50
**Patients’ perceived encouragement from their physicians to achieve SDM**	**94.88**	**87.21**
My physician provided me with detailed information about the disease(s) that I have	97.95	90.44
My physician explained to me the diagnostic and therapeutic decisions that I need to make	98.24	91.42
My physician informed me of different treatment alternatives	88.63	80.09
My physician asked me which treatment alternative I prefer	91.50	82.86
My physician and I reached a consensus on the subsequent treatment process	98.10	91.24
**Informed consent**	**95.69**	**89.68**
My physician explained the medical expenses of special medical care	93.78	87.81
My physician obtained informed consent from me for special medical care	97.60	91.54
**Overall**	**95.30**	**87.86**

PRRs, positive response rates; HPRRs, high positive response rates; n = 2585

Each item in the SDM assessment was rated using a 5-point Likert scale as follows: 1 for “strongly disagree”, 2 for “disagree”, 3 for “neither agree nor disagree”, 4 for “agree” and 5 for “strongly agree”. The SDM measure used in this study had relatively acceptable construct validity and internal reliability (goodness of fit using a confirmatory factor analysis model: SRMR = 0.09, RMSEA = 0.12, GFI = 0.86, AGFI = 0.79; and overall Cronbach’s α = 0.82).

In this study, the percentage of inpatients who rated an item on the SDM scale as “strongly agree” or “agree” was referred to as the positive response rate (PRR) to this item, and the percentage of inpatients who rated an item on the SDM scale as “strongly agree” was referred to as high positive response rate (HPRR) to this item.

#### Inpatient satisfaction scale

Based on our previous inpatient satisfaction scale and consultation with experts in medical care quality, four dimensions with 35 items were used to assess inpatient satisfaction (Additional file 1: Table S2). The four dimensions of the inpatient satisfaction scale were “Facilities and equipment”, “Physician services”, “Nonphysician services” and “Medical care process and effectiveness”. To assess the association of inpatients’ perceived SDM with their satisfaction, overall inpatient satisfaction with medical care, the dimension of “Physician services” (hereafter “physician services”) and two items [“Medical expenses are reasonable” and “I was satisfied with medical care outcomes” (hereafter “medical expenses” and “treatment outcomes”, respectively)] were used.

Each item on the inpatient satisfaction scale was scored using a 5-point Likert scale as follows: 1 for “very dissatisfied”, 2 for “dissatisfied”, 3 for “neither satisfied nor dissatisfied”, 4 for “satisfied” and 5 for “very satisfied”. If an item was irrelevant to a surveyed inpatient, the item was treated as a missing value for this patient. In the analyses, a missing value of an item was replaced by the average score of the item. The percentage of inpatients who rated medical care equal to 5 is referred to as the inpatient high satisfaction rate (HSR).

The psychometric analysis indicated that the inpatient satisfaction measure used in this study had relatively good construct validity based on standard tests of goodness of fit using a confirmatory factor analysis model (GFI = 0.85; AGFI = 0.90; SRMR = 0.04; and RMSEA = 0.05) and had high internal reliability (overall Cronbach’s α = 0.95).

### Statistical analyses

We computed the average PRR and HPRR of the items related to a given aspect as the PRR and HPRR of each aspect of SDM, respectively. We also calculated the average PRR and HPRR if the 13 items on the SDM scale in the survey as a summary statistic, which we refer to as “overall PRR” and “overall HPRR”, respectively. Overall HPRR was computed as the average of all responses received and then computed separately for general hospitals and specialty hospitals, inpatients with cancer and inpatients without cancer, and inpatients who underwent surgery and inpatients who did not undergo surgery.

We computed the HSRs of overall medical care, physician services, medical expenses and treatment outcomes. The HSRs of physician services and overall inpatient care were the average HSRs of the items related to the “Physician services” dimensions and all items on the inpatient satisfaction scale.

To examine whether the hospital type, admission department, inpatient with cancer, and surgery during admission affected the inpatients’ HPRRs to the four aspects of SDM and overall SDM, we applied t-tests and linear regression models.

To illustrate the differences in the adjusted HPRRs between groups of inpatients, we used the coefficients in linear regression models to calculate the adjusted HPRRs while holding all other variables constant at their means and graphically present the relevant predictions.

To test the differences in the inpatients’ overall HSRs and HSRs of physician services, medical expenses and treatment outcomes between the inpatients with or without high level of overall SDM and the four aspects of SDM, we used two-level regression models that accounted for the nesting of individuals within hospitals. In the models, high level of SDM referred to the average HPRRs of each aspect of SDM or overall SDM that were equal to or greater than 80%, while a non-high level of SDM referred to the average HPRRs of each aspect of SDM or overall SDM that were less than 80%. More specifically, two-level linear regression models were used to analyze overall HSR and the HSR of physician services, and the dependent variables were the average HSRs of the items in the “Physician services” dimension and all items on the inpatient satisfaction scale; two-level logistic models were used for the HSRs of medical expenses and treatment outcomes (1: “very satisfied”, 0: others). In addition, high-level SDM, the inpatients’ characteristics (admitting department, inpatient with cancer, surgery during admission, gender, age, residence, education and family monthly income) and the hospital type were used as fixed effects.

The following equations were applied in the two-level mixed linear regression models:

$$\begin{aligned} {\text{HSR}}_{ij} = & \beta_{0j} + \beta_{1} \;{\text{high-level}}\_{\text{of}}\_{\text{SDM}}_{ij} + \beta_{2} \;{\text{Surgery}}_{ij} + \beta_{3} \;{\text{Obstetrics and gynecology}}_{ij} \\ & \quad + \beta_{4} \;{\text{Pediatrics}}_{ij} + \beta_{5} \;{\text{Other}}\_{\text{departments}}_{ij} + \beta_{6} \;{\text{Cancer}}_{ij} + \beta_{{7}} \;{\text{Surgery}}_{ij} \\ & \quad + \beta_{8} \;{\text{Gender}}_{ij} + \beta_{9} \;{\text{Age}}_{ij} + \beta_{10} \;{\text{Residence}}_{ij} + \beta_{11} \;{\text{Education}}_{ij} + \beta_{12} \;{\text{Income}}_{ij} + e_{0j} \\ \end{aligned}$$

$$\beta_{0j} = \gamma_{00} + \gamma_{01} \;{\text{hospital}}\_{\text{type}}_{1j} + \mu_{0j}$$

$$\begin{aligned} {\text{HSR}}_{ij} = & \gamma_{00} + \gamma_{01} \;{\text{hospital}}\_{\text{type}}_{1j} + \beta_{1} \;{\text{high-level}}\_{\text{of}}\_{\text{SDM}}_{ij} + \beta_{2} \;{\text{Surgery}}_{ij} + \beta_{3} \;{\text{Obstetrics and gynecology}}_{ij} \\ & \quad + \beta_{4} \;{\text{Pediatrics}}_{ij} + \beta_{5} \;{\text{Other}}\_{\text{departments}}_{ij} + \beta_{6} \;{\text{Cancer}}_{ij} + \beta_{{7}} \;{\text{Surgery}}_{ij} + \beta_{8} \;{\text{Gender}}_{ij} \\ & \quad + \beta_{9} \;{\text{Age}}_{ij} + \beta_{10} \;{\text{Residence}}_{ij} + \beta_{11} \;{\text{Education}}_{ij} + \beta_{12} \;{\text{Income}}_{ij} + (\mu_{0j} + e_{0j} ) \\ \end{aligned}$$

The following equations were applied in the two-level logistic regression models:

$$\begin{aligned} {\text{In [p}}_{ij} {/}({1} - {\text{p}}_{ij} ){]} = & \beta_{0j} + \beta_{1} \;{\text{high-level}}\_{\text{of}}\_{\text{SDM}}_{ij} + \beta_{2} \;{\text{Surgery}}_{ij} + \beta_{3} \;{\text{Obstetrics and gynecology}}_{ij} \\ & \quad + \beta_{4} \;{\text{Pediatrics}}_{ij} + \beta_{5} \;{\text{Other}}\_{\text{departments}}_{ij} + \beta_{6} \;{\text{Cancer}}_{ij} + \beta_{{7}} \;{\text{Surgery}}_{ij} \\ & \quad + \beta_{8} \;{\text{Gender}}_{ij} + \beta_{9} \;{\text{Age}}_{ij} + \beta_{10} \;{\text{Residence}}_{ij} + \beta_{11} \;{\text{Education}}_{ij} + \beta_{12} \;{\text{Income}}_{ij} \\ \end{aligned}$$

$$\beta_{0j} = \gamma_{00} + \gamma_{01} \;{\text{hospital}}\_{\text{type}}_{1j} + \mu_{0j}$$

$$\begin{aligned} {\text{In[p}}_{ij} {/}({1} - {\text{p}}_{ij} ){]} = & \gamma_{00} + \gamma_{01} \;{\text{hospital}}\_{\text{type}}_{1j} + \beta_{1} \;{\text{high-level}}\_{\text{of}}\_{\text{SDM}}_{ij} + \beta_{2} \;{\text{Surgery}}_{ij} \\ & \quad + \beta_{3} \;{\text{Obstetrics and gynecology}}_{ij} + \beta_{4} \;{\text{Pediatrics}}_{ij} + \beta_{5} \;{\text{Other}}\_{\text{departments}}_{ij} \\ & \quad + \beta_{6} \;{\text{Cancer}}_{ij} + \beta_{{7}} \;{\text{Surgery}}_{ij} + \beta_{8} \;{\text{Gender}}_{ij} + \beta_{9} \;{\text{Age}}_{ij} + \beta_{10} \;{\text{Residence}}_{ij} \\ & \quad + \beta_{11} \;{\text{Education}}_{ij} + \beta_{12} \;{\text{Income}}_{ij} + \mu_{0j} \\ \end{aligned}$$

In the above equations, i = 1, 2,…, n, j = 1, 2,…, m; n is the number of surveyed inpatients, and m is the number of surveyed hospitals.

To determine the appropriateness of the two-level regression models, we examined the empty models of the inpatients’ overall HSR and HSRs to physician services, medical expenses and treatment outcomes. The results showed significant differences in HSRs among hospitals (*P* < 0.001), and the intraclass correlation coefficients (ICC) in the empty models of the inpatients’ overall HSR and HSRs to physician services, medical expenses and treatment outcomes were 0.12, 0.08, 0.09 and 0.09, respectively. Additionally, the − 2 log likelihood, AIC, AICC and BIC in the empty models of the inpatients’ overall HSR and HSRs to physician services and treatment outcomes were greater than those in the non-empty models, and those in the empty model of the inpatients’ HSR to medical expenses were close to those in the non-empty model in this study. Therefore, two-level regression models were appropriate for the analysis of the association between SDM and inpatient satisfaction.

In this study, we also analyzed the variances of the HSRs of overall inpatient care across hospitals and individuals, using the method described by Snijders and Bosker.

This study was approved by the Institutional Review Board of the School of Public Health, Fudan University (IRB#2018-05-0683).

## Results

### Inpatient characteristics

In total, 2585 inpatients in tertiary public hospitals (hereafter “tertiary hospitals”) in Shanghai participated in the study. Among the surveyed inpatients, 69.90% were from general hospitals, 55.86% were aged below 60 years, 52.19% were female, 73.15% had a high school education or below, 37.18% had a family monthly income below 5 thousand yuans, and 60.85% were Shanghai residents. In addition, 15.05% of the surveyed inpatients suffered from cancer, and 44.06% of the inpatients had at least one surgery during hospitalization. The inpatients admitted to internal medicine, surgery, obstetrics and gynecology, pediatrics and other departments accounted for 34.31%, 33.73%, 13.11%, 2.21%, and 16.64% of the sample, respectively (Additional file 1: Table S3).

### Inpatient PRRs and HPRRs to SDM

This study showed that the PRR and the HPPR to overall SDM among the inpatients in the tertiary hospitals in Shanghai were 95.30% and 87.86%, respectively. The HPRRs of the four aspects of SDM (“Patients’ information preference”, “Patients’ active involvement in SDM”, “Patients’ perceived encouragement from their physicians to achieve SDM” and “Informed consent”) were 90.04%, 85.57%, 87.21% and 89.68%, respectively. Although all items related to the four aspects had HPRRs above 80% (80.09–91.54%), “My physician informed me of different treatment alternatives” had the lowest HPRR (80.09%) (Table 1).

### Comparison of HPRRs between different groups of inpatients

This study showed that the inpatients who underwent any surgery during admission had higher HPRRs to “Patients’ information preference”, “Patients’ perceived encouragement from their physicians to achieve SDM” and overall SDM than those who did not undergo surgery during admission (91.49%, 88.96% and 89.23% vs. 88.90%, 85.83% and 86.79%, respectively). The inpatients with cancer had a higher HPRR to “Patients’ perceived encouragement from their physicians to achieve SDM” than those without cancer (90.01% vs. 86.71%) (Table 2).

**Table 2:** Comparison of HPRRs to SDM between different groups of inpatients (%)

Variables	Patients’ information preference	Patients’ active involvement in SDM	Patients’ perceived encouragement from their physicians to achieve SDM	Informed consent	Overall
General hospital					
Yes	89.87	85.04	87.54	89.75	87.84
No	90.43	86.80	86.45	89.51	87.92
Pediatric department					
Yes	88.47	88.25	86.70	88.85	87.80
No	90.07	85.51	87.22	89.70	87.87
Suffering from cancer					
Yes	90.93	86.24	90.01*	91.46	89.57
No	89.88	85.45	86.71	89.36	87.56
Surgery during admission					
Yes	91.49*	86.66	88.96***	90.38	89.23**
No	88.90	84.71	85.83	89.13	86.79

HPRRs, high positive response rates; SDM, shared decision making

*P < 0.05; **P < 0.01; ***P < 0.001

**Fig. 1:**
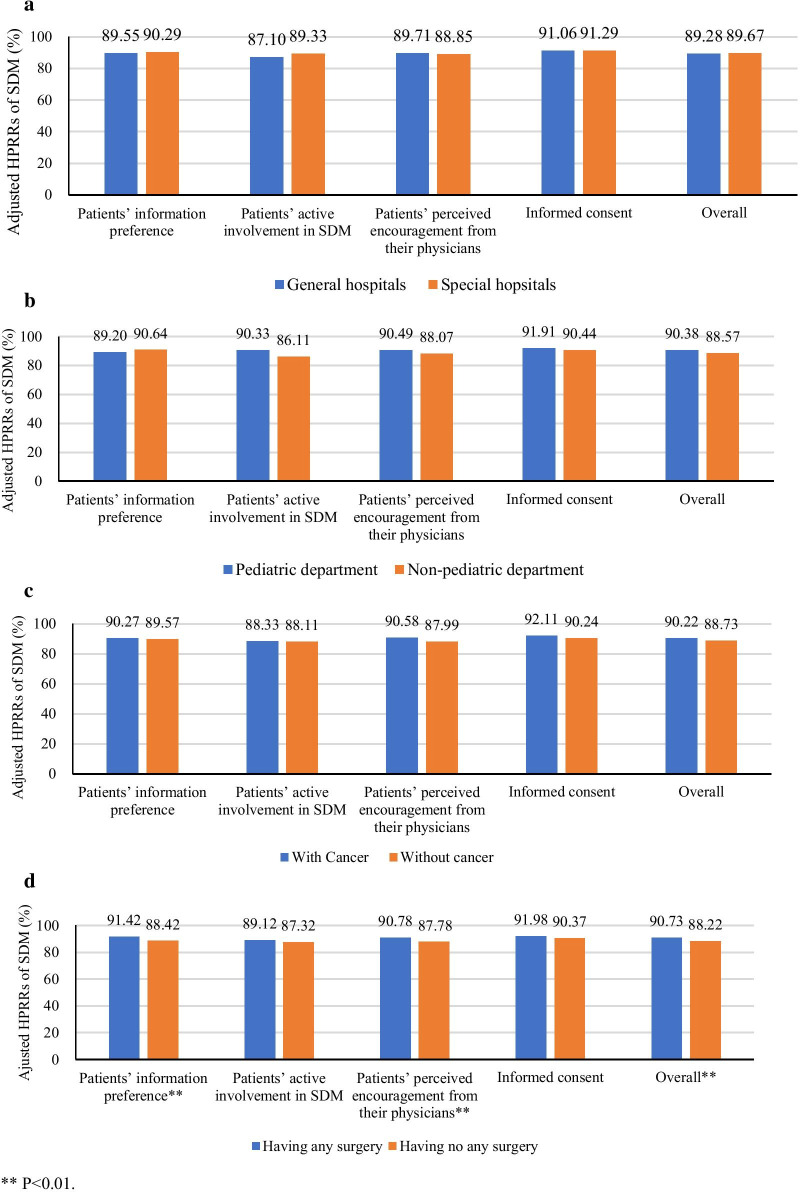
Comparison of the adjusted PRRs to SDM between different groups of inpatients. **P < 0.01

After the application of the linear regression models (Additional file 1: Table S4), the inpatients who underwent surgery had a relatively higher adjusted HPRR to “Patients’ information preference” (91.42%), “Patients’ perceived encouragement from their physicians to achieve SDM” (90.78%) and overall SDM (90.73%) than those who did not undergo surgery (88.42%, 87.78% and 88.22%, respectively). However, there was no significant difference in the adjusted HPRR of overall SDM and the four aspects between the inpatients with or without cancer, between the inpatients in general hospitals or specialty hospitals or between the inpatients in pediatric or non-pediatric departments (Fig. 1).

### Association of SDM with inpatient satisfaction

Among the surveyed inpatients, the HSRs of overall inpatient care, physician services, medical expenses, and treatment outcomes were 87.63%, 91.45%, 61.70% and 83.06%, respectively.

After the adoption of the two-level regression models to account for the nesting of individuals within hospitals and control for other fixed effects, the study showed that the inpatients with a high level of overall SDM had higher adjusted HSRs with overall inpatient care, physician services, medical expenses and treatment outcomes (92.60%, 96.50%, 68.44% and 89.50%, respectively) than those without a high level of overall SDM (67.49%, 71.77%, 35.19% and 57.30%, respectively) (Table 3).

**Table 3:** Comparison of adjusted HSRs between inpatients with or without a high level of SDM (%)

Variables^a^	Overall	Physician services	Medical expenses	Treatment outcomes
Patients' information preference				
High level of SDM	91.53	95.35	66.87	88.56
Non-high level of SDM	70.09	74.80	39.34	58.81
Patients' active involvement in SDM				
High level of SDM	91.84	95.74	67.36	88.39
Non-high level of SDM	72.62	76.89	42.27	64.37
Patients' perceived encouragement from their physicians to achieve SDM				
High level of SDM	92.33	96.23	67.30	88.81
Non-high level of SDM	67.33	71.57	38.03	58.28
Informed consent				
High level of SDM	90.38	94.20	65.65	86.24
Non-high level of SDM	64.79	70.00	30.44	57.16
Overall SDM				
High level of SDM	92.60	96.50	68.44	89.50
Non-high level of SDM	67.49	71.77	35.19	57.30

Two-level mixed linear regression models were used to analyze the overall HSR and HSR with physician services, and the dependent variables were the average HSRs of all items on the inpatient satisfaction scale and the items in the “Physician services” dimension; Two-level logistic models were used to analyze the HSRs with medical expenses and treatment outcomes (1: “very satisfied”, 0: others); two-level regression models were used to calculate the adjusted HSRs of the inpatients with or without positive responses to SDM while controlling for the hospital type, admitting department, inpatient characteristics (age, sex, residence, education and income), inpatient with or without cancer, and having surgery; all p-values of the t-tests in the models were < 0.0001; SDM, shared decision making; HSRs, high positive response rates

^a^The group of inpatients with a high level of SDM was defined as the inpatient group in which all inpatients had an average HPRR to an aspect or overall equal to or greater than 80%, while the group of inpatients with a non-high level of SDM was referred as the inpatient group in which inpatients had an average HPRR to an aspect or overall less than 80%

Regarding to inpatient satisfaction with overall inpatient care, physician services, medical expenses and treatment outcomes, the greatest differences in the adjusted HSRs between the inpatients with or without a high level of all four aspects of SDM were found in the “Medical expenses” item (25–36 percentage points). Among the 4 aspects of SDM, the greatest differences in the adjusted HSRs were observed in the “Informed consent” aspect (24–36 percentage points) (Table 3 and Additional file 1: Table S5–S9).

### Variation in inpatient satisfaction across hospitals

The two-level regression models of the HSRs showed that the inpatient HSRs with overall inpatient care, physician services and treatment outcomes in the specialty hospitals were significantly lower than those in the general hospitals after controlling for a high level of overall SDM and other individual factors (Additional file 1: Table S5). When analyzing the variances using the method described by Snijders and Bosker, the results showed that 46.22% of the variances in the HSRs with overall inpatient care across the hospitals were attributed to the hospital type (general hospitals vs. specialty hospitals), while 30.80% of the variances in the HSRs with overall inpatient care across individuals were attributed to the Level-1 model.

## Discussion

### Relatively high overall SDM but lower informing regarding alternatives

In recent years, due to rapid innovation and more uncertainty in medical care, hospitals have become increasingly aware of the need to deliver “patient-centered” care and have paid increasing attention to physician–patient communication and SDM [30, 31]. In this study, we found that the PRR and the HPPR of overall SDM among the inpatients in tertiary hospitals in Shanghai were 95.30% and 87.86%, respectively. The HPRRs to “Patients’ active involvement in SDM” and “Patients’ perceived encouragement from their physicians to achieve SDM” among the inpatients in tertiary hospitals in Shanghai were 85.57% and 87.21%, respectively, which are close to those reported in other studies [87% of patients with newly diagnosed, localized prostate cancer who reported being actively involved in treatment decision making [32] and the mean SDM-Q-9 score (68, full score = 100) and median CollaboRATE score (93, full score = 100) of outpatients with vascular malformations [33]]. In recent years, the action plan called for by the central and local governments to further improve medical service and train staff in physician–patient communication skills [34, 35] has facilitated the implementation of SDM in tertiary hospitals in Shanghai.

However, similar to another study in cardiology that revealed that fewer patients reported “some” or “a lot of” discussions regarding the advantages and disadvanges of treatment options (88% and 58% regarding transcatheter aortic valve replacement and 78% and 49% regarding surgical aortic valve replacement, respectively) [36], we found that the HPRR to “My physician informed me of different treatment alternatives” was relatively low (80.09%). The basis for patients’ involvement in treatment decisions is patients’ full understanding of different treatment alternatives [37], which is also the basis for patients signing informed consent for surgery. If patients are not informed about alternatives, it is difficult for them to know whether the treatment recommended by their physicians will be the most beneficial.

### SDM affected by preference and medical condition

In this study, we found that inpatients who underwent any surgery during admission had better perceptions of SDM than those who did not undergo any surgery. These findings persisted when we used linear regression models to control for other factors. Complex clinical decisions with higher risks and more critical health outcomes in the patients’ survey [38] may have led to both patients and physicians having a higher preference for SDM. Moreover, informed consent before surgery is required not only as a legal doctrine but also as patient-centered care [39]. SDM in inpatients undergoing surgery helps physicians understand patients’ values, preferences, and needs and helps patients understand the benefits and risks of surgical alternatives to reduce physician–patient conflict and protect patient interests [40, 41]. These factors may explain the better SDM in the inpatients who underwent any surgery.

Similar to other studies [42, 43], our study found that inpatients with cancer had a significantly higher HPRR to “Patients’ perceived encouragement from their physicians to achieve SDM” than those without cancer (90.01% vs. 86.71%). The reason for this finding could be that the guidelines for communication with cancer patients strongly recommend that physicians clarify the treatment goals to support their patients’ hope and understanding, provide information regarding all available treatment options and the advantages and disadvantages of each option, and respect the patients’ treatment autonomy [44]. However, there was no significant difference in the adjusted HPRRs in SDM between the inpatients with or without cancer.

### Inpatient satisfaction improved by SDM

Our study revealed that the inpatients with a high level of responses regarding overall SDM had much higher adjusted HSRs with physician services, medical expenses, treatment outcomes and overall inpatient care (96.50%, 68.44%, 89.50% and 92.60%, respectively) than those without a high level of overall responses (71.77%, 35.19%, 57.30% and 67.49%, respectively).

Furthermore, our study revealed the unique finding that SDM had a greater influence on inpatient satisfaction with medical expenses and that informed consent had a greater influence on inpatient satisfaction with tertiary hospitals in Shanghai. In tertiary hospitals, medical expenses can be a high burden for patients, and treatment selection is an important determinant of patient outcomes. A higher level of SDM can reduce medical costs of care [4, 45], facilitate discussion regarding the benefits, risks and costs (including considerable out-of-pocket treatments) of options [36, 46] and enhance informed consent for complex clinical decisions [47]. Therefore, better SDM (especially informed consent) in tertiary hospitals in Shanghai can improve patient satisfaction with medical expenses, treatment outcomes, physician services and overall patient satisfaction.

### Increase in patient satisfaction at the hospital level

Patients’ experience with medical care is an important aspect of the quality of care. The measurement of patients’ experiences and the dissemination of measurement results can help identify weaknesses in medical care, improve the medical care quality and promote patient choice [48, 49]. In total, 46.22% of the variances in the HSRs with overall inpatient care across the hospitals were attributed to the hospital type (general hospitals vs. specialty hospitals), and the inpatients in the specialty hospitals had lower satisfaction with overall medical care than those in the general hospitals, highlighting the importance of hospital-level commitment to increasing patient satisfaction. To facilitate improvement in patient satisfaction in tertiary hospitals, especially specialty hospitals, patients’ experiences or satisfaction and SDM should be measured, the results should be disseminated, and incentives should be provided for delivering “patient-centered” care.

## Limitations

All hospitals included in our study were located in Shanghai, which is among the most developed areas in China, and our findings may not be generalizable to hospitals in other areas of China. In addition, the inpatients surveyed in our study had not been discharged from the hospitals, although they had completed their main medical care (e.g., surgeries or therapeutic procedures). Therefore, systemic bias may exist in the patient selection. However, the overall satisfaction rate of the inpatients who were still hospitalized did not significantly differ from that of inpatients who were surveyed during their hospital discharge process (96.71% vs. 97.01%, *P* > 0.05) in our contemporaneous survey. The inpatients surveyed during their hospital discharge process were not surveyed with regard to SDM. Therefore, the bias from our inpatient survey might not be significant. Moreover, many factors contribute to patients’ perceived SDM and patient satisfaction [50–52]. We used linear regression models and two-level regression models to minimize some potential confounders (e.g., socioeconomic factors), but other confounders were not considered in this study. In addition, the SDM measure used in this study did not have high construct validity. All above limitations may affect our results to some extent.

## Conclusions

The inpatient PRRs and HPRRs to SDM in public tertiary hospitals in Shanghai are relatively high overall but lower to information regarding alternatives. Furthermore, SDM can be affected by the SDM preferences of both patients and physicians and the medical condition. Patient satisfaction with physician services, medical expenses, treatment outcomes and overall inpatient care can be improved through better SDM, especially by providing information regarding treatment alternatives to patients and obtaining informed consent when treatments or procedures are high risk, expensive or involve considerable out-of-pocket costs, and hospital-level commitment.

## Supplementary information

**Additional file 1.** Supplementary tables.

## Data Availability

The dataset used in the current study is available from the corresponding author upon reasonable request.

## Abbreviations

SDM: Shared decision making
PICS: Perceived Involvement in Care Scale
SDM-Q-9: 9-Item Shared Decision Making Questionnaire
SDM-Q-Doc: Shared Decision Making Questionnaire-physician version
PRR: Positive response rate
HPRR: High positive response rate
SR: Satisfaction rate
HSR: High satisfaction rate
