# Supplementary material for: Association of shared decision making with inpatient satisfaction: a cross-sectional study
Source: BMC Med Inform Decis Mak. 2021 Jan 25;21:25. doi: 10.1186/s12911-021-01385-1 (PMC7831242; doi:10.1186/s12911-021-01385-1)
Supplement: Supplementary file 1 — Additional file 1. Supplementary tables. [file 12911_2021_1385_MOESM1_ESM.docx]

# Additional file.

# Table S1: Scores of the importance of measuring SDM and sources of the items.

| Preliminary aspects and items | | Mean | STD | Scale based | Items included |
| --- | --- | --- | --- | --- | --- |
| **Patients’ information preference** | |  |  |  |  |
|  | I should be provided with more information about my illness when I become sicker. | 6.68 | 2.46 | API | no |
|  | I should sufficiently understand the effects of the disease(s) that I have on my health. | 7.44 | 2.52 | API | yes |
|  | Even if the news about my health and the disease(s) that I have is bad, I should be well informed. | 6.26 | 2.30 | API | no |
|  | The physician should explain to me the purposes of the test(s) and/or examination(s). | 7.80 | 1.91 | API | yes |
|  | My physician should provide information to me only when I ask for it. | 6.16 | 2.15 | API | no |
|  | It is very important for me to know all side effects of my medication. | 6.82 | 1.84 | API | no |
|  | I believe that getting information about the disease(s) is as important as getting information about the treatment. | 7.32 | 2.50 | API | yes |
|  | When there is more than one method to treat a problem, my physician should tell me about each one. | 6.48 | 2.38 | API | no |
| **Patients’ active involvement in SDM** | |  |  |  |  |
|  | I asked my physician to explain the treatment alternatives and process in detail. | 7.64 | 2.08 | PICS | yes |
|  | I asked my physician to provide treatment recommendations to me. | 7.24 | 1.92 | PICS | yes |
|  | I asked for great detail about my medical symptoms. | 7.32 | 2.39 | PICS | yes |
|  | I asked my physician many questions about my medical symptoms. | 6.21 | 2.52 | PICS | no |
|  | I suggested a certain type of medical treatment to my physician. | 5.76 | 2.20 | PICS | no |
|  | I insisted on a particular type of test or treatment for "better treatment". | 5.08 | 2.22 | PICS | no |
|  | I expressed doubts about the tests or treatment that my physician recommended. | 6.29 | 1.90 | PICS | no |
|  | I gave my opinion (agreement or disagreement) about the types of tests or treatments that my doctor ordered. | 6.28 | 2.09 | PICS | no |
| **Patients’ perceived encouragement from their physicians to achieve SDM** | |  |  |  |  |
|  | My physician provided me with detailed information about the disease(s) that I have | 7.72 | 2.28 | PICS/CollaboRATE | yes |
|  | My physician explained to me the diagnostic and therapeutic decisions that I need to make. | 7.72 | 2.01 | SDM-Q-9/SDM-Q-Doc | yes |
|  | My physician asked me about my willingness to be involved in making the decision. | 6.84 | 2.56 | SDM-Q-9/SDM-Q-Doc | no |
|  | My physician informed me of different treatment alternatives. | 7.40 | 1.85 | SDM-Q-9/SDM-Q-Doc | yes |
|  | My physician helped me understand relevant medical information. | 6.80 | 2.18 | SDM-Q-9/SDM-Q-Doc | no |
|  | My physician asked me which treatment alternative I prefer. | 7.28 | 2.01 | SDM-Q-9/SDM-Q-Doc//CollaboRATE | yes |
|  | My physician and I weighed the different treatment options together (benefits and risks of the treatment options). | 6.54 | 2.30 | SDM-Q-9/SDM-Q-Doc | no |
|  | My physician and I selected a treatment option together. | 6.42 | 2.30 | SDM-Q-9/SDM-Q-Doc | no |
|  | My physician and I reached a consensus on the subsequent treatment process. | 7.00 | 2.04 | SDM-Q-9/SDM-Q-Doc | yes |
| **Informed consent** | |  |  |  |  |
|  | My physician explained the medical expenses of special medical care (such as medical care that was not on the reimbursement list of basic medical insurance, or tests, examinations and drugs with a high price). | 8.30 | 1.72 | - | yes |
|  | My physician obtained informed consent from me for special medical care (such as traumatic examinations, expensive tests or examinations, and surgery). | 8.35 | 1.80 | - | yes |

# Table S2: Dimensions and items on the inpatient satisfaction scale.

|  | Dimensions and items |
| --- | --- |
| **Facilities and equipment** | |
|  | The hospital had clear guide signs. |
|  | The ward was clean, quiet, well ventilated and suitable in temperature. |
|  | The hospital provided clean and convenient articles, equipment and facilities for daily use. |
|  | The ward was quiet at night. |
| **Physician services** | |
|  | The physician was courteous. |
|  | The physician made ward rounds every day (including holidays). |
|  | The senior physician made ward rounds regularly. |
|  | The physician carefully listened to my condition description and actively asked questions. |
|  | The physician carefully conducted physical examinations. |
|  | The physician adequately explained the results of tests and exams, diagnoses, medication and precautions to me. |
|  | The physician explained detailed future effects of the illness to me. |
|  | The physician provided detailed information regarding alternative treatments and suggested a treatment plan. |
|  | The physician answered my questions in a way that I could understand. |
|  | The physician considered my opinions and conditions when making treatment decisions. |
|  | The physician paid attention to my privacy protection during the exams and treatment. |
| **Nonphysician services** | |
|  | The nurses were courteous. |
|  | The nurse actively provided a welcome and introduction, indicated the inpatient area and provided other information when I was admitted. |
|  | The nurse made ward rounds frequently, communicated with me actively, understood my demands and answered my questions. |
|  | The nurse came to my bedside immediately after I pressed the call button. |
|  | The nurse provided me with appropriate health education. |
|  | The nurse paid attention to protecting my privacy during nursing. |
|  | The nurse was skilled in practice. |
|  | The nurse, volunteer or social worker could provide timely, appropriate psychological counseling. |
|  | The staff members in the hospital admission and discharge office were courteous and answered questions. |
|  | The medical technician was courteous and answered questions. |
|  | The attendant and security personnel were courteous and answered questions. |
| **Medical care process and effectiveness** | |
|  | I was satisfied with the food provided by the hospital. |
|  | The therapeutic diet was appropriate. |
|  | The hospital charge list was clearly publicized, and the hospital charge bills were detailed and clear. |
|  | The medical expenses were reasonable. |
|  | The hospital admission queue was well ordered. |
|  | The admission procedure was convenient. |
|  | I was satisfied with the medical care outcomes. |
|  | If needed, I would choose to receive inpatient care in this hospital again. |
|  | I am willing to recommend that friends and family members receive medical care in this hospital. |

# Table S3: Demographics of the surveyed inpatients.

|  | Variables | No. | Percent (%) |  |  | Variables | No. | Percent (%) |
| --- | --- | --- | --- | --- | --- | --- | --- | --- |
| Hospital type | |  |  |  | Department | |  |  |
|  | General hospital | 1807 | 69.90 |  |  | Internal medicine | 887 | 34.31 |
|  | Specialty hospital | 778 | 30.10 |  |  | Surgery | 872 | 33.73 |
|  | Total | 2585 | 100.00 |  |  | Obstetrics and gynecology | 339 | 13.11 |
| Age (years) | |  |  |  |  | Pediatrics | 57 | 2.21 |
|  | ≥60 | 1141 | 44.14 |  |  | Other | 430 | 16.64 |
|  | <60 | 1444 | 55.86 |  |  | Total | 2585 | 100.00 |
|  | Total | 2585 | 100.00 |  | Patients with cancer | |  |  |
| Gender | |  |  |  |  | Yes | 389 | 15.05 |
|  | Male | 1236 | 47.81 |  |  | No | 2196 | 84.95 |
|  | Female | 1349 | 52.19 |  |  | Total | 2585 | 100.00 |
|  | Total | 2585 | 100.00 |  | Having surgery | |  |  |
| Education | |  |  |  |  | Yes | 1139 | 44.06 |
|  | High school or below | 1891 | 73.15 |  |  | No | 1446 | 55.94 |
|  | College or above | 694 | 26.85 |  |  | Total | 2585 | 100.00 |
|  | Total | 2585 | 100.00 |  |  |  |  |  |
| Family monthly income | |  |  |  |  |  |  |  |
|  | Low income (<5000 yuan) | 961 | 37.18 |  |  |  |  |  |
|  | High income (≥5000 yuan) | 1624 | 62.82 |  |  |  |  |  |
|  | Total | 2585 | 100.00 |  |  |  |  |  |
| Residence | |  |  |  |  |  |  |  |
|  | Shanghai | 1573 | 60.85 |  |  |  |  |  |
|  | Non-Shanghai | 1012 | 39.15 |  |  |  |  |  |
|  | Total | 2585 | 100.00 |  |  |  |  |  |

# Table S4: Linear regression models of the factors influencing SDM^†^.

|  | Parameters | Patients' information preference | |  | Patients' active involvement in SDM^‡^ | |  | Patients' perceived encouragement from their physicians | |  | Informed consent | |  | Overall SDM | |
| --- | --- | --- | --- | --- | --- | --- | --- | --- | --- | --- | --- | --- | --- | --- | --- |
|  |  | β | P |  | β | P |  | β | P |  | β | P |  | β | P |
| Intercept | | 0.8787 | <.0001 |  | 0.8041 | <.0001 |  | 0.8267 | <.0001 |  | 0.8497 | <.0001 |  | 0.8370 | <.0001 |
| Hospital type (control=General hospital) | |  |  |  |  |  |  |  |  |  |  |  |  |  |  |
|  | Specialty hospital (1: yes, 0: no) | 0.0073 | 0.5040 |  | 0.0223 | 0.0748 |  | -0.0086 | 0.4410 |  | 0.0022 | 0.8358 |  | 0.0039 | 0.6777 |
| Departments (control=Internal medicine) | |  |  |  |  |  |  |  |  |  |  |  |  |  |  |
|  | Surgery (1: yes, 0: no) | -0.0152 | 0.2020 |  | 0.0044 | 0.7424 |  | 0.0006 | 0.9604 |  | -0.0074 | 0.5273 |  | -0.0034 | 0.7367 |
|  | Obstetrics and gynecology (1: yes, 0: no) | -0.0029 | 0.8678 |  | 0.0311 | 0.1134 |  | 0.0285 | 0.1047 |  | 0.0125 | 0.4614 |  | 0.0194 | 0.1851 |
|  | Pediatrics (1: yes, 0: no) | -0.0144 | 0.6748 |  | 0.0423 | 0.2781 |  | 0.0243 | 0.4862 |  | 0.0147 | 0.6620 |  | 0.0180 | 0.5347 |
|  | Others (1: yes, 0: no) | -0.0173 | 0.2192 |  | -0.0371 | 0.0206 |  | -0.0058 | 0.6860 |  | 0.0019 | 0.8920 |  | -0.0145 | 0.2246 |
| Patients with cancer (1: yes, 0: no) | | 0.0071 | 0.5907 |  | 0.0022 | 0.8813 |  | 0.0259 | 0.0524 |  | 0.0187 | 0.1484 |  | 0.0150 | 0.1781 |
| Having surgery (1: yes, 0: no) | | 0.0300 | 0.0024 |  | 0.0180 | 0.1087 |  | 0.0300 | 0.0027 |  | 0.0162 | 0.0951 |  | 0.0251 | 0.0026 |
| Age (1:≥60, 0: <60) | | 0.0002 | 0.4741 |  | 0.0003 | 0.2934 |  | 0.0005 | 0.0685 |  | 0.0003 | 0.1847 |  | 0.0003 | 0.1100 |
| Male (1: yes, 0: no) | | 0.0084 | 0.3992 |  | 0.0058 | 0.6105 |  | 0.0136 | 0.1799 |  | 0.0055 | 0.5734 |  | 0.0093 | 0.2683 |
| Residence (1: Shanghai, 0: Non-Shanghai) | | 0.0034 | 0.7418 |  | 0.0159 | 0.1752 |  | 0.0001 | 0.9918 |  | 0.0165 | 0.1038 |  | 0.0070 | 0.4208 |
| Education (1: College or above,0: High school  or below) | | 0.0204 | 0.0741 |  | 0.0273 | 0.0356 |  | 0.0057 | 0.6208 |  | 0.0131 | 0.2448 |  | 0.0152 | 0.1155 |
| Family monthly income (1: <5000 yuan),  0: ≥5000 yuan)) | | -0.0190 | 0.0581 |  | 0.0026 | 0.8176 |  | -0.0134 | 0.1903 |  | 0.0094 | 0.3420 |  | -0.0075 | 0.3786 |
| -2 Res Log Pseudolikelihood | | -109.6 |  |  | 544.8 |  |  | -30.6 |  |  | -201.3 |  |  | -968.3 |  |
| AIC | | -107.6 |  |  | 546.8 |  |  | -28.6 |  |  | -199.3 |  |  | -966.3 |  |
| Z value | | 35.86 |  |  | 35.86 |  |  | 35.86 |  |  | 35.86 |  |  | 35.86 |  |
| P | | <.0001 |  |  | <.0001 |  |  | <.0001 |  |  | <.0001 |  |  | <.0001 |  |

† The dependent variables in the models were the average HPRR of the items of a given aspect or the average HPRR of the 13 items on the SDM scale; HPRRs: high positive response rates;

‡ SDM: shared decision making.

# Table S5: Two-level regression models of the association of overall SDM with inpatient HSR^†^.

|  |  | Overall | |  | Physician services | |  | Medical expenses | |  | Treatment outcomes | |
| --- | --- | --- | --- | --- | --- | --- | --- | --- | --- | --- | --- | --- |
|  | Parameters | β | P |  | β | P |  | β | P |  | β | P |
| Intercept | | 0.6721 | <.0001 |  | 0.7074 | <.0001 |  | 0.4133 | <.0001 |  | 0.5937 | <.0001 |
| High level of overall SDM (1: yes, 0: no) ‡ | | 0.2510 | <.0001 |  | 0.2473 | <.0001 |  | 0.3326 | <.0001 |  | 0.3221 | <.0001 |
| Hospital type (control: General hospital) | |  |  |  |  |  |  |  |  |  |  |  |
|  | Specialty hospital | -0.0404 | 0.0205 |  | -0.0340 | 0.0181 |  | -0.0386 | 0.3417 |  | -0.0621 | 0.0136 |
| Departments (control: Internal medicine) | |  |  |  |  |  |  |  |  |  |  |  |
|  | Surgery (1: yes, 0: no) | -0.0036 | 0.6510 |  | -0.0028 | 0.7493 |  | -0.0381 | 0.1042 |  | -0.0014 | 0.9399 |
|  | Obstetrics and gynecology (1: yes, 0: no) | -0.0093 | 0.4925 |  | -0.0126 | 0.3799 |  | 0.0870 | 0.0251 |  | -0.0022 | 0.9379 |
|  | Pediatrics (1: yes, 0: no) | -0.0142 | 0.6261 |  | -0.0270 | 0.3724 |  | 0.0214 | 0.7942 |  | -0.0837 | 0.1583 |
|  | Others (1: yes, 0: no) | 0.0274 | 0.0098 |  | 0.0173 | 0.1269 |  | 0.0081 | 0.7899 |  | 0.0179 | 0.4308 |
| Age (1:≥60, 0: <60) | | -0.0002 | 0.3159 |  | 0.0000 | 0.8852 |  | -0.0023 | <.0001 |  | -0.0006 | 0.1626 |
| Male (1: yes, 0: no) | | 0.0078 | 0.2467 |  | 0.0058 | 0.4306 |  | 0.0301 | 0.1247 |  | -0.0087 | 0.5597 |
| Residence (1: Shanghai, 0: Non-Shanghai) | | 0.0086 | 0.2260 |  | 0.0116 | 0.1364 |  | 0.0216 | 0.2985 |  | 0.0014 | 0.9273 |
| Education (1: College or above, 0: High school or below) | | 0.0034 | 0.6607 |  | -0.0040 | 0.6422 |  | 0.0514 | 0.0239 |  | 0.0251 | 0.1481 |
| Family monthly income (1: <5000 yuans, 0: ≥5000 yuans) | | 0.0064 | 0.3434 |  | 0.0027 | 0.7192 |  | 0.0189 | 0.3400 |  | -0.0069 | 0.6500 |
| Having surgery (1: yes, 0: no) | | 0.0186 | 0.0420 |  | 0.0220 | 0.0028 |  | 0.0253 | 0.1978 |  | 0.0556 | 0.0002 |
| In patients with Cancer (1: yes, 0: no) | | 0.0181 | 0.0074 |  | 0.0196 | 0.0488 |  | 0.0380 | 0.1527 |  | 0.0117 | 0.5604 |
| -2 Res Log Pseudolikelihood | | -2158.00 |  |  | -1708.30 |  |  | 3344.46 |  |  | 1960.72 |  |
| AIC | | -2154.00 |  |  | -1704.30 |  |  | 3348.46 |  |  | 1964.72 |  |
| AICC | | -2154.00 |  |  | -1704.30 |  |  | 3348.46 |  |  | 1964.72 |  |
| BIC | | -2150.30 |  |  | -1700.60 |  |  | 3352.16 |  |  | 1968.42 |  |

† Two-level mixed linear regression models were used to analyze the overall HSR and HSR with physician services, and the dependent variables were the average HSRs of all items on the inpatient satisfaction scale and the items in the “Physician services” dimension; two-level logistic models were used to analyze the HSRs with medical expenses and treatment outcomes (1: “very satisfied”, 0: others); SDM: shared decision making; high satisfaction rates (HSRs); HSRs: the percentage of inpatients who were “very satisfied” with their medical care;

‡ If the average HPRR of the overall SDM was equal to or greater than 80%, it was coded as "1"; otherwise, the average HPRR was coded as "0".

# Table S6: Two-level regression models of the association of patients' information preference with inpatient HSR^†^.

|  |  | Overall | |  | Physician services | |  | Medical expenses | |  | Treatment outcomes | |
| --- | --- | --- | --- | --- | --- | --- | --- | --- | --- | --- | --- | --- |
|  | Parameters | β | P |  | β | P |  | β | P |  | β | P |
| Intercept | | 0.6765 | <.0001 |  | 0.7174 | <.0001 |  | 0.4264 | <.0001 |  | 0.5840 | <.0001 |
| High level of "Patients’ information preference" (1: yes, 0: no) | | 0.2144 | <.0001 |  | 0.2055 | <.0001 |  | 0.2753 | <.0001 |  | 0.2975 | <.0001 |
| Hospital type (control: General hospital) | |  |  |  |  |  |  |  |  |  |  |  |
|  | Specialty hospital | -0.0361 | 0.0502 |  | -0.0300 | 0.0562 |  | -0.0329 | 0.4158 |  | -0.0577 | 0.0242 |
| Departments (control: Internal medicine) | |  |  |  |  |  |  |  |  |  |  |  |
|  | Surgery (1: yes, 0: no) | 0.0093 | 0.2752 |  | 0.0098 | 0.2873 |  | -0.0215 | 0.3660 |  | 0.0154 | 0.3962 |
|  | Obstetrics and gynecology (1: yes, 0: no) | 0.0034 | 0.8120 |  | -0.0007 | 0.9649 |  | 0.1034 | 0.0085 |  | 0.0138 | 0.6347 |
|  | Pediatrics (1: yes, 0: no) | 0.0036 | 0.9065 |  | -0.0093 | 0.7720 |  | 0.0474 | 0.5681 |  | -0.0549 | 0.3623 |
|  | Others (1: yes, 0: no) | 0.0194 | 0.0835 |  | 0.0099 | 0.4114 |  | -0.0029 | 0.9243 |  | 0.0075 | 0.7453 |
| Age (1:≥60, 0: <60) | | 0.0000 | 0.8771 |  | 0.0002 | 0.4257 |  | -0.0020 | 0.0005 |  | -0.0003 | 0.4288 |
| Male (1: yes, 0: no) | | 0.0131 | 0.0645 |  | 0.0109 | 0.1592 |  | 0.0369 | 0.0629 |  | -0.0023 | 0.8783 |
| Residence (1: Shanghai, 0: Non-Shanghai) | | 0.0100 | 0.1850 |  | 0.0130 | 0.1125 |  | 0.0237 | 0.2604 |  | 0.0035 | 0.8244 |
| Education (1: College or above, 0: High school or below) | | 0.0023 | 0.7834 |  | -0.0052 | 0.5647 |  | 0.0501 | 0.0300 |  | 0.0220 | 0.2089 |
| Family monthly income (1: <5000 yuans, 0: ≥5000 yuans) | | 0.0106 | 0.1409 |  | 0.0068 | 0.3800 |  | 0.0242 | 0.2274 |  | -0.0003 | 0.9846 |
| Having surgery (1: yes, 0: no) | | 0.0138 | 0.0527 |  | 0.0180 | 0.0203 |  | 0.0201 | 0.3142 |  | 0.0489 | 0.0013 |
| In patients with Cancer (1: yes, 0: no) | | 0.0252 | 0.0091 |  | 0.0262 | 0.0122 |  | 0.0468 | 0.0821 |  | 0.0194 | 0.3423 |
| -2 Res Log Pseudolikelihood | | -1875.40 |  |  | -1447.50 |  |  | 3414.71 |  |  | 2020.81 |  |
| AIC | | -1871.40 |  |  | -1443.50 |  |  | 3418.71 |  |  | 2024.81 |  |
| AICC | | -1871.40 |  |  | -1443.50 |  |  | 3418.71 |  |  | 2024.82 |  |
| BIC | | -1867.70 |  |  | -1439.80 |  |  | 3422.41 |  |  | 2028.51 |  |

† Two-level mixed linear regression models were used to analyze the overall HSR and HSR with physician services, and the dependent variables were the average HSRs of all items on the inpatient satisfaction scale and the items in the “Physician services” dimension; two-level logistic models were used to analyze the HSRs with medical expenses and treatment outcomes (1: “very satisfied”, 0: others); SDM: shared decision making; high satisfaction rates (HSRs); HSRs: the percentage of inpatients who were “very satisfied” with their medical care;

‡ If the average HPRR of "Patients’ information preference" was equal to or greater than 80%, it was coded as "1"; otherwise, the average HPRR was coded as "0".

# Table S7: Two-level regression models of the association of patients' active involvement in SDM with inpatient HSR^†^.

|  |  | Overall | |  | Physician services | |  | Medical expenses | |  | Treatment outcomes | |
| --- | --- | --- | --- | --- | --- | --- | --- | --- | --- | --- | --- | --- |
|  | Parameters | β | P |  | β | P |  | β | P |  | β | P |
| Intercept | | 0.7110 | <.0001 |  | 0.7476 | <.0001 |  | 0.4689 | <.0001 |  | 0.6516 | <.0001 |
| High level of "Patients’ active involvement in SDM" (1: yes, 0: no) | | 0.1922 | <.0001 |  | 0.1885 | <.0001 |  | 0.2509 | <.0001 |  | 0.2402 | <.0001 |
| Hospital type (control: General hospital) | |  |  |  |  |  |  |  |  |  |  |  |
|  | Specialty hospital | -0.0411 | 0.0329 |  | -0.0351 | 0.0308 |  | -0.0398 | 0.3497 |  | -0.0640 | 0.0207 |
| Departments (control: Internal medicine) | |  |  |  |  |  |  |  |  |  |  |  |
|  | Surgery (1: yes, 0: no) | 0.0047 | 0.5863 |  | 0.0052 | 0.5774 |  | -0.0274 | 0.2506 |  | 0.0095 | 0.6068 |
|  | Obstetrics and gynecology (1: yes, 0: no) | -0.0052 | 0.7185 |  | -0.0096 | 0.5297 |  | 0.0919 | 0.0202 |  | 0.0009 | 0.9771 |
|  | Pediatrics (1: yes, 0: no) | -0.0020 | 0.9499 |  | -0.0160 | 0.6214 |  | 0.0392 | 0.6397 |  | -0.0692 | 0.2645 |
|  | Others (1: yes, 0: no) | 0.0289 | 0.0112 |  | 0.0196 | 0.1047 |  | 0.0100 | 0.7474 |  | 0.0219 | 0.3530 |
| Age (1:≥60, 0: <60) | | 0.0000 | 0.9443 |  | 0.0002 | 0.4836 |  | -0.0020 | 0.0004 |  | -0.0004 | 0.3847 |
| Male (1: yes, 0: no) | | 0.0091 | 0.2053 |  | 0.0070 | 0.3645 |  | 0.0319 | 0.1088 |  | -0.0071 | 0.6446 |
| Residence (1: Shanghai, 0: Non-Shanghai) | | 0.0037 | 0.6243 |  | 0.0067 | 0.4152 |  | 0.0151 | 0.4753 |  | -0.0052 | 0.7478 |
| Education (1: College or above, 0: High school or below) | | 0.0033 | 0.6888 |  | -0.0041 | 0.6500 |  | 0.0509 | 0.0276 |  | 0.0242 | 0.1736 |
| Family monthly income (1: <5000 yuans, 0: ≥5000 yuans) | | 0.0001 | 0.9865 |  | -0.0033 | 0.6755 |  | 0.0105 | 0.6020 |  | -0.0139 | 0.3707 |
| Having surgery (1: yes, 0: no) | | 0.0213 | 0.0031 |  | 0.0251 | 0.0012 |  | 0.0300 | 0.1324 |  | 0.0605 | <.0001 |
| In patients with Cancer (1: yes, 0: no) | | 0.0286 | 0.0033 |  | 0.0295 | 0.0049 |  | 0.0511 | 0.0578 |  | 0.0239 | 0.2485 |
| -2 Res Log Pseudolikelihood | | -1818.40 |  |  | -1424.90 |  |  | 3422.23 |  |  | 2089.00 |  |
| AIC | | -1814.40 |  |  | -1420.90 |  |  | 3426.23 |  |  | 2093.00 |  |
| AICC | | -1814.40 |  |  | -1420.90 |  |  | 3426.23 |  |  | 2093.00 |  |
| BIC | | -1810.70 |  |  | -1417.20 |  |  | 3429.93 |  |  | 2096.70 |  |

† Two-level mixed linear regression models were used to analyze the overall HSR and HSR with physician services, and the dependent variables were the average HSRs of all items on the inpatient satisfaction scale and the items in the “Physician services” dimension; two-level logistic models were used to analyze the HSRs with medical expenses and treatment outcomes (1: “very satisfied”, 0: others); SDM: shared decision making; high satisfaction rates (HSRs); HSRs: the percentage of inpatients who were “very satisfied” with their medical care;

‡ If the average HPRR of "Patients’ active involvement in SDM" was equal to or greater than 80%, it was coded as "1"; otherwise, the average HPRR was coded as "0".

# Table S8: Two-level regression models of the association of patients' perceived encouragement from their physicians with inpatient HSR^†^.

|  |  | Overall | |  | Physician services | |  | Medical expenses | |  | Treatment outcomes | |
| --- | --- | --- | --- | --- | --- | --- | --- | --- | --- | --- | --- | --- |
|  | Parameters | β | P |  | β | P |  | β | P |  | β | P |
| Intercept | | 0.6700 | <.0001 |  | 0.7022 | <.0001 |  | 0.4252 | <.0001 |  | 0.5894 | <.0001 |
| High level of "Patients’ perceived encouragement from their physicians" (1: yes, 0: no) | | 0.2354 | <.0001 |  | 0.2365 | <.0001 |  | 0.2928 | <.0001 |  | 0.3053 | <.0001 |
| Hospital type (control: General hospital) | |  |  |  |  |  |  |  |  |  |  |  |
|  | Specialty hospital | -0.0314 | 0.0859 |  | -0.0256 | 0.0862 |  | -0.0272 | 0.5144 |  | -0.0518 | 0.0455 |
| Departments (control: Internal medicine) | |  |  |  |  |  |  |  |  |  |  |  |
|  | Surgery (1: yes, 0: no) | 0.0020 | 0.8133 |  | 0.0025 | 0.7778 |  | -0.0305 | 0.1986 |  | 0.0058 | 0.7453 |
|  | Obstetrics and gynecology (1: yes, 0: no) | -0.0097 | 0.4903 |  | -0.0128 | 0.3847 |  | 0.0879 | 0.0254 |  | -0.0019 | 0.9465 |
|  | Pediatrics (1: yes, 0: no) | -0.0112 | 0.7079 |  | -0.0210 | 0.4987 |  | 0.0298 | 0.7208 |  | -0.0725 | 0.2301 |
|  | Others (1: yes, 0: no) | 0.0196 | 0.0736 |  | 0.0101 | 0.3840 |  | -0.0022 | 0.9432 |  | 0.0092 | 0.6887 |
| Age (1:≥60, 0: <60) | | -0.0001 | 0.6633 |  | 0.0001 | 0.7531 |  | -0.0021 | 0.0002 |  | -0.0004 | 0.2672 |
| Male (1: yes, 0: no) | | 0.0093 | 0.1781 |  | 0.0072 | 0.3335 |  | 0.0324 | 0.1014 |  | -0.0068 | 0.6516 |
| Residence (1: Shanghai, 0: Non-Shanghai) | | 0.0115 | 0.1187 |  | 0.0142 | 0.0722 |  | 0.0250 | 0.2332 |  | 0.0045 | 0.7763 |
| Education (1: College or above, 0: High school or below) | | 0.0054 | 0.4976 |  | -0.0019 | 0.8278 |  | 0.0542 | 0.0184 |  | 0.0275 | 0.1149 |
| Family monthly income (1: <5000 yuans, 0: ≥5000 yuans) | | 0.0080 | 0.2531 |  | 0.0044 | 0.5627 |  | 0.0205 | 0.3063 |  | -0.0043 | 0.7732 |
| Having surgery (1: yes, 0: no) | | 0.0183 | 0.0084 |  | 0.0222 | 0.0031 |  | 0.0265 | 0.1817 |  | 0.0563 | 0.0002 |
| In patients with Cancer (1: yes, 0: no) | | 0.0148 | 0.1157 |  | 0.0157 | 0.1211 |  | 0.0341 | 0.2034 |  | 0.0065 | 0.7459 |
| -2 Res Log Pseudolikelihood | | -2014.30 |  |  | -1616.30 |  |  | 3395.88 |  |  | 2004.22 |  |
| AIC | | -2010.30 |  |  | -1612.30 |  |  | 3399.88 |  |  | 2008.22 |  |
| AICC | | -2010.30 |  |  | -1612.30 |  |  | 3399.88 |  |  | 2008.22 |  |
| BIC | | -2006.60 |  |  | -1608.60 |  |  | 3403.58 |  |  | 2011.92 |  |

† Two-level mixed linear regression models were used to analyze the overall HSR and HSR with physician services, and the dependent variables were the average HSRs of all items on the inpatient satisfaction scale and the items in the “Physician services” dimension; two-level logistic models were used to analyze the HSRs with medical expenses and treatment outcomes (1: “very satisfied”, 0: others); SDM: shared decision making; high satisfaction rates (HSRs); HSRs: the percentage of inpatients who were “very satisfied” with their medical care;

‡ If the average HPRR of "Patients’ perceived encouragement from their physicians to achieve SDM" was equal to or greater than 80%, it was coded as "1"; otherwise, the average HPRR was coded as "0".

# Table S9: Two-level regression models of the association of informed consent with inpatient HSR^†^.

|  |  | Overall | |  | Physician services | |  | Medical expenses | |  | Treatment outcomes | |
| --- | --- | --- | --- | --- | --- | --- | --- | --- | --- | --- | --- | --- |
|  | Parameters | β | P |  | β | P |  | β | P |  | β | P |
| Intercept | | 0.6377 | <.0001 |  | 0.6823 | <.0001 |  | 0.3577 | <.0001 |  | 0.5818 | <.0001 |
| High level of "Informed consent" (1: yes, 0: no) | | 0.2558 | <.0001 |  | 0.2420 | <.0001 |  | 0.3521 | <.0001 |  | 0.2908 | <.0001 |
| Hospital type (control: General hospital) | |  |  |  |  |  |  |  |  |  |  |  |
|  | Specialty hospital | -0.0368 | 0.0599 |  | -0.0306 | 0.0656 |  | -0.03429 | 0.4187 |  | -0.0578 | 0.0353 |
| Departments (control: Internal medicine) | |  |  |  |  |  |  |  |  |  |  |  |
|  | Surgery (1: yes, 0: no) | 0.0071 | 0.4086 |  | 0.0076 | 0.4156 |  | -0.0242 | 0.3072 |  | 0.0122 | 0.5091 |
|  | Obstetrics and gynecology (1: yes, 0: no) | -0.0006 | 0.9687 |  | -0.0043 | 0.7795 |  | 0.0990 | 0.0121 |  | 0.0086 | 0.7728 |
|  | Pediatrics (1: yes, 0: no) | -0.0057 | 0.8545 |  | -0.0189 | 0.5634 |  | 0.0372 | 0.6564 |  | -0.0696 | 0.2636 |
|  | Others (1: yes, 0: no) | 0.0191 | 0.0922 |  | 0.0096 | 0.4299 |  | -0.0037 | 0.9051 |  | 0.0078 | 0.7427 |
| Age (1:≥60, 0: <60) | | -0.0002 | 0.4207 |  | 0.0000 | 0.9780 |  | -0.0023 | <.0001 |  | -0.0005 | 0.2071 |
| Male (1: yes, 0: no) | | 0.0113 | 0.1123 |  | 0.0093 | 0.2324 |  | 0.0347 | 0.0794 |  | -0.0041 | 0.7913 |
| Residence (1: Shanghai, 0: Non-Shanghai) | | 0.0063 | 0.4097 |  | 0.0093 | 0.2600 |  | 0.0181 | 0.3888 |  | -0.0015 | 0.9272 |
| Education (1: College or above, 0: High school or below) | | 0.0043 | 0.6018 |  | -0.0029 | 0.7497 |  | 0.0519 | 0.0241 |  | 0.0261 | 0.1446 |
| Family monthly income (1: <5000 yuans, 0: ≥5000 yuans) | | -0.0007 | 0.9274 |  | -0.0039 | 0.6211 |  | 0.0091 | 0.6511 |  | -0.0146 | 0.3509 |
| Having surgery (1: yes, 0: no) | | 0.0247 | 0.0006 |  | 0.0285 | 0.0003 |  | 0.0345 | 0.0822 |  | 0.0649 | <.0001 |
| In patients with Cancer (1: yes, 0: no) | | 0.0226 | 0.0198 |  | 0.0240 | 0.0228 |  | 0.0429 | 0.1104 |  | 0.0173 | 0.4062 |
| -2 Res Log Pseudolikelihood | | -1836.40 |  |  | -1405.50 |  |  | 3403.84 |  |  | 2118.25 |  |
| AIC | | -1832.40 |  |  | -1401.50 |  |  | 3407.84 |  |  | 2122.25 |  |
| AICC | | -1832.40 |  |  | -1401.50 |  |  | 3407.85 |  |  | 2122.25 |  |
| BIC | | -1828.70 |  |  | -1397.80 |  |  | 3411.54 |  |  | 2125.95 |  |

† Two-level mixed linear regression models were used to analyze the overall HSR and HSR with physician services, and the dependent variables were the average HSRs of all items on the inpatient satisfaction scale and the items in the “Physician services” dimension; two-level logistic models were used to analyze the HSRs with medical expenses and treatment outcomes (1: “very satisfied”, 0: others); SDM: shared decision making; high satisfaction rates (HSRs); HSRs: the percentage of inpatients who were “very satisfied” with their medical care;

‡ If the average HPRR of "Informed consent" was equal to or greater than 80%, it was coded as "1"; otherwise, the average HPRR was coded as "0".
